# Draft genome sequences of *Pantoea agglomerans* and *Pantoea vagans* isolates associated with termites

**DOI:** 10.1186/s40793-016-0144-z

**Published:** 2016-03-01

**Authors:** Marike Palmer, Pieter de Maayer, Michael Poulsen, Emma T. Steenkamp, Elritha van Zyl, Teresa A. Coutinho, Stephanus N. Venter

**Affiliations:** Department of Microbiology and Plant Pathology and the Genome Research Institute, University of Pretoria, Pretoria, 0002 South Africa; DST-NRF Centre of Excellence in Tree Health Biotechnology, Forestry and Agricultural Biotechnology Institute, University of Pretoria, Pretoria, 0002 South Africa; Centre for Microbial Ecology and Genomics, University of Pretoria, Pretoria, 0002 South Africa; Department of Biology, Centre for Social Evolution, Section for Ecology and Evolution, University of Copenhagen, Univeritetsparken 15, 2100 Copenhagen East, Denmark

**Keywords:** *Pantoea*, Bacteria, Insect, Symbiosis

## Abstract

The genus *Pantoea* incorporates many economically and clinically important species. The plant-associated species, *Pantoea agglomerans* and *Pantoea vagans,* are closely related and are often isolated from similar environments. Plasmids conferring certain metabolic capabilities are also shared amongst these two species. The genomes of two isolates obtained from fungus-growing termites in South Africa were sequenced, assembled and annotated. A high number of orthologous genes are conserved within and between these species. The difference in genome size between *P. agglomerans* MP2 (4,733,829 bp) and *P. vagans* MP7 (4,598,703 bp) can largely be attributed to the differences in plasmid content. The genome sequences of these isolates may shed light on the common traits that enable *P. agglomerans* and *P. vagans* to co-occur in plant- and insect-associated niches.

## Introduction

The bacterial genus *Pantoea* contains several economically important plant pathogens, as well as strains of clinical importance [10]. Amongst the plant pathogens, *Pantoea ananatis*, with its broad host range (e.g. onion, eucalyptus and pineapple) and *P. stewartii* subsp. *stewartii*, the causal agent of Stewart’s wilt on maize, are the best known. The human pathogens include species such as *P. septica* and *P. brenneri* [9], although some plant-associated species have also been isolated from immuno-compromised patients [12, 17]. *P. agglomerans* and *P. vagans* are most commonly isolated from similar ecological niches, including both plant and insect hosts [41].

Three plasmids (pPag1, pPag2 and pPag3) were identified in the genome of the biocontrol strain *P. vagans* C9-1 [45] and it is thought that the presence of these plasmids may play a role in the physiological and ecological functioning of this strain. The plasmid, pPag1, codes for sucrose metabolism, while the plasmid, pPag2, harbours genes for an antimicrobial peptide and sorbitol utilization [33, 46]. The megaplasmid pPag3 belongs to the LPP-1 plasmids conserved among all sequenced *Pantoea* sppecies to date and carries genes involved in pigment production, thiamine biosynthesis and maltose metabolism [19, 46]. In contrast to *P. vagans*, some strains of *P. agglomerans* are also known to induce galls on *Gypsophila* spp., beet (*Beta vulgaris*), Douglas fir (*Pseudotsuga menziesii*) and *Wisteria* spp. [6, 37]. This ability has been linked to a genomic island that encodes a Type III secretion system and pPath plasmid genes involved in the biosynthesis of the plant hormones, indole-3-acetic acid and cytokinins [6]. *P. agglomerans* strains have also been shown to cause opportunistic infections in humans [15, 18].

In this study we summarize the features of a *P. agglomerans* (Mn107) and a *P. vagans* (Mn109) that were isolated from two different colonies of the fungus-growing termite *Macrotermes natalensis* in South Africa, and provide an overview of the draft genome sequences and annotations for these two strains. The genome sequences provide some understanding of the shared genomic features that could be linked to their survival in similar environments and the unique features that characterise the species.

## Organism information

### Classification and features

**Fig. 1:**
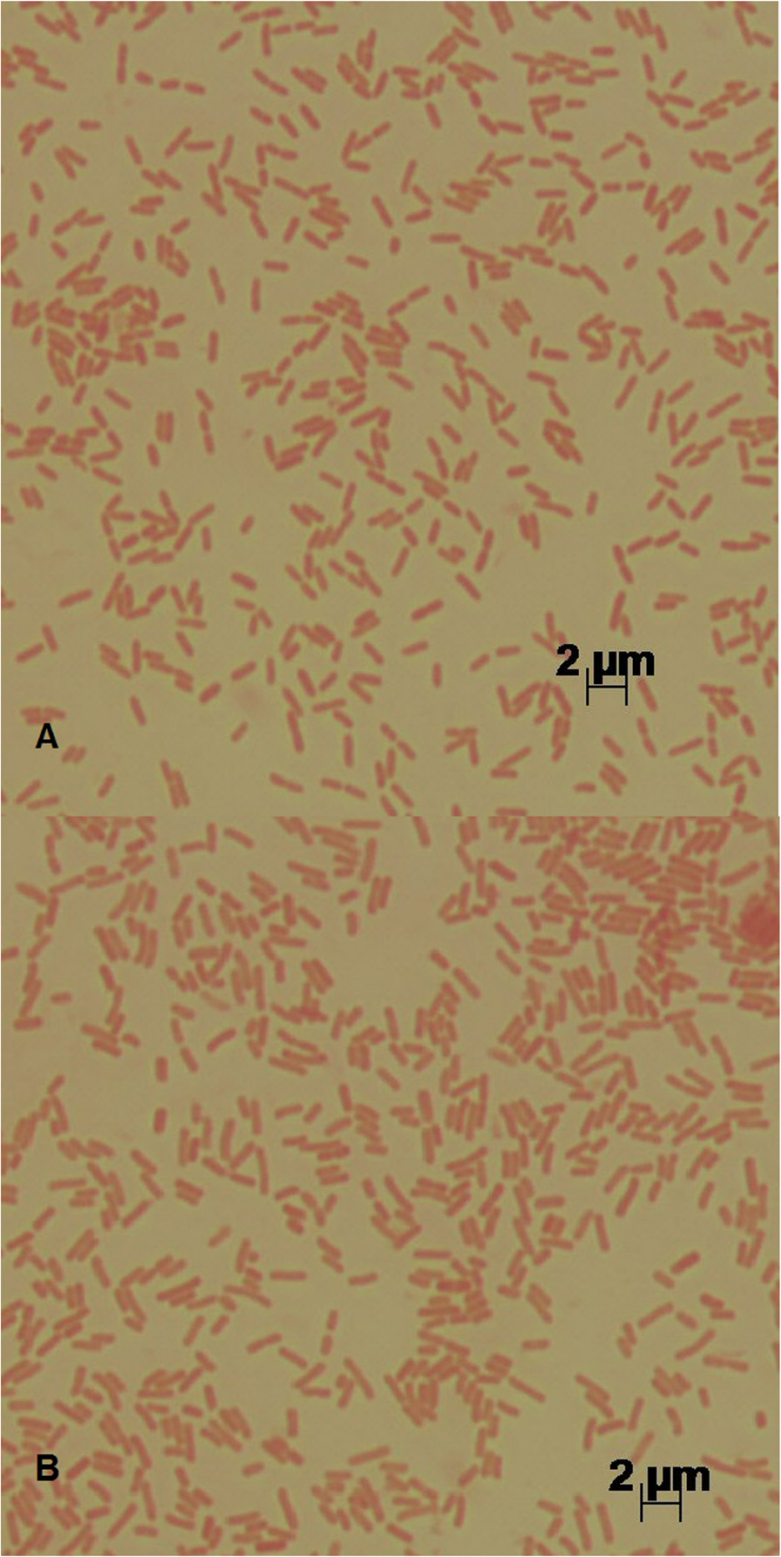
Photomicrographs of source organisms. The source organisms for **a** *P. agglomerans* MP2 and of **b** *P. vagans* MP7, stained with safranin

Both *P. agglomerans* MP2 (LMG 29065) and *P. vagans* MP7 (LMG 29064) are members of the *Enterobacteriaceae* in the class *Gammaproteobacteria*, and are thus Gram-negative, motile, non-spore-forming, rods (Fig. 1, Table 1). After incubation on Luria-Bertani agar (10 g tryptone, 5 g yeast extract, 5 g NaCl, and X g agar per litre) at 28 °C for 24 h, colonies of *P. agglomerans* MP2 and *P. vagans* MP7 are yellow, convex and round with entire margins.

**Table 1:** Classification and general features of *P. agglomerans* MP2 and *P. vagans* MP7

MIGS ID	Property	*Pantoea agglomerans* MP2	Evidence code^a^	*Pantoea vagans* MP7	Evidence code^a^
	Classification	Bacteria	NAS [25]	Bacteria	NAS [25]
		*Proteobacteria*	NAS [23]	*Proteobacteria*	NAS [23]
		*Gammaproteobacteria*	NAS [24, 51]	*Gammaproteobacteria*	NAS [24, 51]
		*Enterobacteriaceae*	NAS [42, 44]	*Enterobacteriaceae*	NAS [42, 44]
		*Enterobacteriales*	NAS [25]	*Enterobacteriales*	NAS [25]
		*Pantoea*	NAS [9, 26]	*Pantoea*	NAS [9, 26]
		*Pantoea agglomerans*	NAS [26, 39]	*Pantoea vagans*	NAS [10]
	Gram stain	Negative	NAS [26]	Negative	NAS [10]
	Cell shape	Straight rods	NAS [26]	Short rods	NAS [10]
	Motility	Motile	NAS [26]	Motile	NAS [10]
	Sporulation	Non-sporeforming	NAS [26]	Non-sporeforming	NAS [10]
	Temperature range	Mesophile	NAS [26]	Mesophile	NAS [10]
	Optimum temperature	30 °C	NAS [54]	30 °C	NAS [54]
	pH range; Optimum	4 - 8; 5–6	IDA	4 - 9; 5 -6	IDA
	Carbon source	D-Glucose, L-arabinose, D-galactose, maltose, D-mannitol, D-mannose, L-rhamnose, sucrose, trehalose, D-xylose	NAS [54]	Malonic acid, L-ornithine, D-glucose, L-arabinose, D-ribose, D-galactose, sucrose, maltose	NAS [10]
	Energy source	Chemoorganotroph	NAS [54]	Chemoorganotroph	NAS [54]
	Terminal electron receptor	Not available		Not available	
MIGS-6	Habitat	Termite	IDA	Termite	IDA
MIGS-6.3	Salinity	Not available		Not available	
MIGS-22	Oxygen requirement	Facultative anaerobic	NAS [54]	Facultative anaerobic	NAS [54]
MIGS-15	Biotic relationship	Potential termite symbiont		Potential termite symbiont	
MIGS-14	Pathogenicity	Not available		Not available	
MIGS-4	Geographic location	Pretoria, South Africa		Mookgophong, South Africa	
MIGS-5	Sample collection	January 2010		January 2010	
MIGS-4.1 MIGS-4.2	Latitude – Longitude	S25 43 45.6 E28 14 09.9		S24 40 30.5 E28 47 50.4	
MIGS-4.3	Depth	N/A		N/A	
MIGS-4.4	Altitude	1344 m		1046 m	

*IDA* Inferred from Direct Assay, *TAS* Traceable Author Statement (i.e., a direct report exists in the literature), *NAS* Non-traceable Author Statement (i.e., not directly observed for the living, isolated sample, but based on a generally accepted property for the species, or anecdotal evidence). These evidence codes are derived from the Gene Ontology project

^a^Evidence codes

**Fig. 2:**
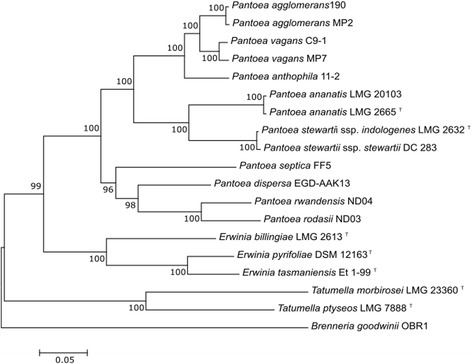
Maximum likelihood phylogenetic tree indicating the phylogenetic relationship of sequenced isolates. The maximum likelihood (ML) tree was constructed from an alignment of concatenated *atpD, carA, gyrB, infB, recA* and *rpoB* gene sequences [57]. The tree was constructed with Mega 6 [49] using the general time reversible (GTR) model [36] with the estimation of the proportion of invariable sites and gamma distribution. Bootstrap support values were calculated from 1000 bootstrap replicates. Several strains (including type strains; indicated with “^T^”) of *Pantoea* sppecies for which genome sequences are publicly available were included in the analysis [Genbank Accessions: *P. agglomerans* 190 [26]: GCA_000731125.1, *P. vagans* C9-1 [10]: GCA_000148935.1, *P. anthophila* 11–2 [10]: GCA_000969395.1, *P. stewartii* subsp. *indologenes* LMG 2632^T^ [38]: GCA_000757405.1, *P. stewartii* subsp. *stewartii* DC283 [38]: GCA_000248395.2, *P. ananatis* LMG 2665 ^T^ [38]: GCA_000710035.1, *P. ananatis* LMG 20103 [38]: GCA_000025405.2, *P. septica* FF5 [9]: GCA_000612605.1, *P. dispersa* EGD-AAK13 [26]: GCA_000465555.2, *P. rodasii* ND03 [11]: GCA_000801085.1, *P. rwandensis* ND04 [11]:GCA_000759475.1]. Type strains of species of the sister genera *Tatumella* [*Tatumella ptyseos* LMG 7888 T [31, 52]: GCA_000439895.1 and *Tatumella morbirosei* LMG 23360 T [31]: GCA_000757425.2 (Genbank Accessions)] and *Erwinia* [44, 55], [*Erwinia billingiae* LMG 2613 T [39]: GCA_000196615.1, *Erwinia pyrifoliae* DSM 12163 [34]: GCA_000026985.1, *Erwinia tasmaniensis* Et-99: GCA_000026185.1 (Genbank Accessions)], for which genome sequences are available, were also included. *Brenneria goodwinii* OBR-1 [GCA_001049335.1 (Genbank Accession)] was used as outgroup

The 16S rRNA gene sequences of the enteric bacteria tend to provide insufficient resolution and the phylogenetic relationships of *P. agglomerans* MP2 and *P. vagans* MP7 were therefore inferred with multi-locus sequence analysis. This analysis included closely related members in the genus *Pantoea* with available genome sequences, and was based on partial nucleotide sequences of four protein coding genes (i.e., *atpD*, *carA, gyrB*, *infB, recA* and *rpoB*) [57]. Our results showed that *P. agglomerans* and *P. vagans* group as sister-species (Fig. 2).

The two isolates (strain codes: MP2 = Mn109-1w1C and MP7 = Mn107-old1M) were isolated from *Macrotermes natalensis* termite mounds in 2010. The surface of worker termite was rinsed using phospate buffer saline and MP2 was isolated from the rinsate, which was inoculated directly onto chitin medium (4 g chitin, 0.7 g K_2_HPO_4_, 0.3 g KH_2_PO_4_, 0.5 g MgSO_4_.5H_2_O, 0.01 g FeSO_4_.7H_2_0, 0.001 g ZnSO_4_, 0.001 g MnCl_2_, and 20 g of agar per litre), while MP7 was isolated from fungus comb ground in PBS and inoculated onto Carboxymethyl cellulose medium (10 g carboxymethyl cellulose and 20 g agar per litre). Isolates were streaked onto Yeast Malt Extract Agar medium (4 g yeast extract, 10 g malt extract, 4 g D-glucose and 20 g bacteriological agar per litre), and once in pure culture, they were stored in 10 % glycerol at −20 °C. The specificity and possible role of associations between fungus-growing termites and the two *Pantoea* isolates have not been determined, but the abundance of members of the *Enterobacteriaceae* in both fungus-growing termite guts [40] and fungus combs [4] suggests the possibility of a specific association.

## Genome sequencing information

### Genome project history

The genomes of both isolates were sequenced using the Illumina platform. Velvet [56] and Mauve [16] were employed for the assembly of the genomes and annotations were done using the Rapid Annotation using Subsystem Technology [5] and WebMGA. The genomes will remain as high quality drafts and are available from the National Center for Biotechnology Information (Tables 2 and 3). The Whole Genome Shotgun projects have been deposited at DDBJ/EMBL/GenBank under the accessions JPKQ00000000 and JPKP00000000, respectively. The versions described in this paper are version JPKQ00000000.1 and JPKP00000000.1.

**Table 2:** Project information

MIGS ID	Property	*P. agglomerans* MP2	*P. vagans* MP7
MIGS-31	Finishing quality	High-quality draft	High-quality draft
MIGS-28	Libraries used	500 bp	500 bp
MIGS-29	Sequencing platforms	Illumina HiSeq mate-pair	Illumina HiSeq mate-pair
MIGS-31.2	Fold coverage	179 ×	184 ×
MIGS-30	Assemblers	Velvet	Velvet
MIGS-32	Gene calling method	RAST	RAST
	Genbank ID	JPKQ00000000.1	JPKP00000000.1
	Genbank Date of Release	23/9/2014	23/9/2014
	GOLD ID	Gp0099200	Gp0099199
	BIOPROJECT	PRJNA254768	PRJNA254769
MIGS-13	Source material identifier	SAMN02905153	SAMN02905155
	Project relevance	Potential termite symbiont	Potential termite symbiont

**Table 3:** Summary of the genomes

	Label	Size (Mb)	Topology	INSDC identifier	RefSeq ID
*Pantoea agglomerans* MP2	Chromosome 1	3988.2	circular	JPKQ0100001-13	NZ_JPKQ01000001.1-13.1
	Plasmid 1	184.9	circular	JPKQ01000014	NZ_JPKQ01000014.1
	Plasmid 2	292.9	circular	JPKQ01000015	NZ_JPKQ01000015.1
	Plasmid 3	531.5	circular	JPKQ01000016	NZ_JPKQ01000016.1
*Pantoea vagans* MP7	Chromosome 1	3913.1	circular	JPKP01000001-6	NZ_JPKP01000001.1-6.1
	Plasmid 1	176.9	circular	JPKP01000007	NZ_JPKP01000007.1
	Plasmid 2	508.6	circular	JPKP01000008	NZ_JPKP01000008.1

### Growth conditions and genomic DNA preparation

Pure cultures of the MP2 and MP7 isolates that were initially grown at 28 °C on YMEA plates was then cultured in Luria-Bertani broth (10 g tryptone, 5 g yeast extract, and 5 g NaCl per litre). DNA was subsequently extracted from the cultures using the Qiagen DNeasy blood and tissue kit (Qiagen, CA). DNA quality was assessed using a NanoDrop™ spectrophotometer.

### Genome sequencing and assembly

**Fig. 3:**
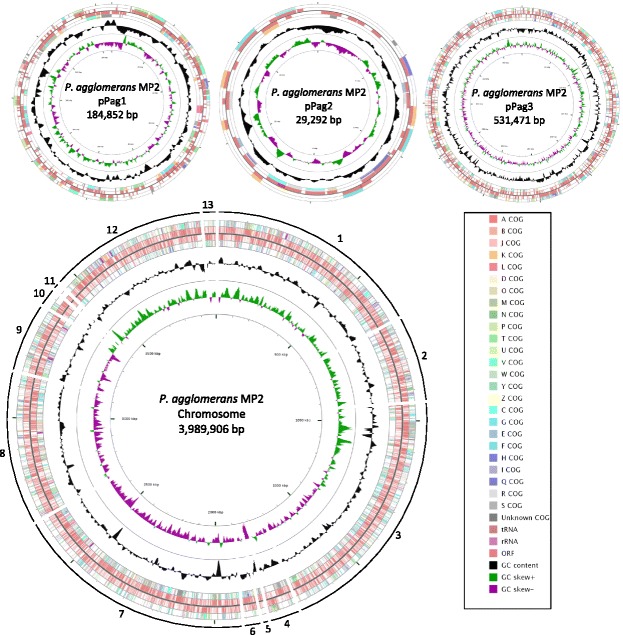
The genome structure of *P. agglomerans* MP2. The genome consists of 1 chromosome and 3 plasmids. The order of the contigs was based on the publicly available complete genome sequence of *P. vagans* C9-1 [45]. The sizes of the contigs varied significantly with the smallest being just below 5 kbp (contig 5) and the largest being just less than 800 kbp (contig 3). The open-reading frames (ORFs) for the forward and reverse strands are indicated in the inner tracks, flanked by the COG classes associated with the respective ORFs. The GC content across the genome is indicated in black, with the GC skew (calculated as [G-C/G + C]) indicated in green and purple, respectively [48]

**Fig. 4:**
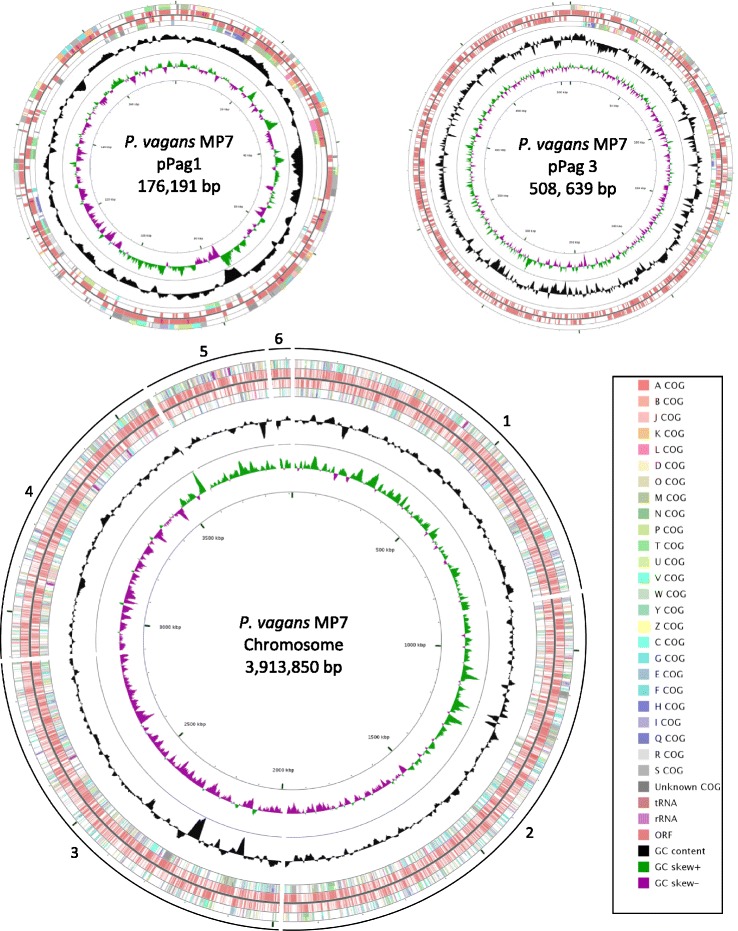
The genome structure of *P. vagans* MP7. The genome consists of 1 chromosome and 2 plasmids. The order of the contigs was based on the complete genome sequence of *P. vagans* C9-1 which is publicly available [45]. The contigs varied in size with the largest (contig 2) being approximately 1,010 kbp and the smallest (contig 6) being just below 50 kbp. The predicted ORFs are indicated in the inner tracks and are flanked with the COG classes associated with each of the ORFs. The GC content of the various regions within the genome is indicated in black, with the GC skew indicated in green and purple [48]

The genomes of the two isolates were sequenced using mate-paired Illumina sequencing using the HiSeq Platform at the Beijing Genomics Institute. Libraries with an insert size of 500 bp were generated and sequence lengths of 90 bp in both directions were obtained. After filtering out reads with >10 % Ns and/or 25–35 bases of low quality (≤Q20), and removing adapter and duplication contamination as well as trimming read ends, approximately 850 Mb of sequence data remained per isolate. The sequence reads were assembled using Velvet [56] and the sequencing and assembly metrics are given in Table 2. Contigs generated in this way were further assembled into contiguous scaffolds by alignment against the closest complete genomes, based on BLAST, of *P. vagans* C9-1 [45] and the draft genome of *Pantoea* sp. SL1-M5 [1] using the progressive Mauve algorithm in Mauve 2.3.1 [16]. The final genomes had coverage of *ca.* 180 ×, where that of MP2 consisted of 16 contigs and that of MP7 consisted of 8 contigs (Figs. 3 and 4).

### Genome annotation

The genomes were annotated using the RAST pipeline [5]. RAST initiated the annotation by predicting RNA molecules, followed by an initial gene prediction and placing of the genome into phylogenetic context. The most closely related genomes were used to assess protein families using FIGfams (i.e., sets of protein sequences that are similar along their full length and that likely represent isofunctional homologs). The remaining genes were then assessed against the FIGfam database [5], followed by metabolic reconstruction. The number of protein-coding genes with functional predictions was thus based on the subsystem technology of RAST.

Both genomes were also subjected to analysis on WebMGA, where comparisons to the Clusters of Orthologous Genes [50] and Protein family (pfam) databases [7] were performed with rpsblast [2]. Signal peptide prediction and transmembrane helix prediction for the protein-coding genes in the genomes were performed using Phobius [32]. CRISPR repeats were detected using the CRISPRs database [29] (Table 4).

**Table 4:** Nucleotide content and gene count levels of the genomes

Attribute	*Pantoea agglomerans* MP2 (total)		*Pantoea vagans* MP7 (total)	
	Value	% of total^a^	Value	% of total^a^
Genome size (bp)	4,733,829	100 %	4,598,703	100 %
DNA coding (bp)	4,043,819	85.4 %	3,948,783	85.9 %
DNA G + C (bp)	2,614,812	55.2 %	2,541,699	55.3 %
DNA scaffolds	16	-	8	-
Total genes^b^	4449	-	4277	-
Protein coding genes	4355	100 %	4181	100 %
RNA genes	94	2.2 %	91	2.2 %
Pseudo genes	-	-	2	0.1 %
Genes in internal clusters	-	-	-	-
Genes with function prediction	3470	79.7 %	3351	80.1 %
Genes assigned to COGs	3686	84.6 %	3608	86.3 %
Genes with Pfam domains	2124	48.8 %	2064	49.4 %
Genes with signal peptides	810	18.6 %	768	18.4 %
Genes with transmembrane helices	927	21.3 %	906	21.7 %
CRISPR repeats	4	0.09 %	3	0.07 %

^a^The percentage of total is based on either the size of the genome in base pairs or the total number of protein coding genes in the annotated genome

^b^Also includes pseudogenes and other genes

## Genome properties

The total genomes of *P. agglomerans* MP2 and *P. vagans* MP7 were 4,733,829 bp and 4,598,703 bp in size, respectively (Table 4; Figs. 3 and 4). The *P. agglomerans* MP2 genome includes three closed plasmids which show high sequence similarity and synteny to pPag1, pPag2 and pPag3 of *P. vagans* C9-1. The genome of *P. vagans* MP7 on the other hand incorporates only copies of pPag1 and pPag3. The pPag2-harbored herbicolin biosynthetic locus of *P. vagans* C9-1 is absent from the genomes of both MP2 and MP7 [33], while the pPATH pathogenicity island [37] is likewise absent from both strains. For *P. agglomerans* MP2, 85.4 % (4,043,819 bp) of the genome coded for 4,449 genes. Of these, 4,355 genes were protein-coding. For *P. vagans* MP7, 85.9 % (3,948,783 bp) of the genome coded for 4181 protein-coding genes. The majority of protein-coding genes had functional predictions using both RAST annotations and the COG database (Table 4). A high number of genes code for proteins that are involved in metabolism (COG codes C, G, E, F, H, I, P and Q) with fewer genes involved in all other classes (Table 5).

**Table 5:** Number and proportion of genes associated with 25 COG functional categories

	*P. agglomerans* MP2		*P. vagans* MP7		
Code	Value	% of total^a^	Value	% of total^a^	Description
J	196	4.50 %	194	4.54 %	Translation
A	1	0.02 %	2	0.05 %	RNA processing and modification
K	358	8.22 %	331	7.74 %	Transcription
L	147	3.38 %	137	3.20 %	Replication, recombination and repair
B	-	-	-	-	Chromatin structure and dynamics
D	42	0.96 %	42	1.00 %	Cell cycle control, Cell division, chromosome partitioning
Y	-	-	-	-	Nuclear structure
V	48	1.10 %	50	1.17 %	Defence mechanisms
T	228	5.24 %	225	5.26 %	Signal transduction mechanisms
M	239	5.49 %	242	5.66 %	Cell wall/membrane biogenesis
N	90	2.07 %	92	2.15 %	Cell motility
Z	-	-	-	-	Cytoskeleton
W	-	-	-	-	Extracellular structures
U	78	1.79 %	82	1.92 %	Intracellular trafficking and secretion
O	137	3.15 %	133	3.11 %	Posttranslational modification, protein turnover, chaperones
C	209	4.80 %	206	4.82 %	Energy production and conversion
G	395	9.07 %	378	8.84 %	Carbohydrate transport and metabolism
E	405	9.30 %	405	9.47 %	Amino acid transport and metabolism
F	96	2.20 %	100	2.34 %	Nucleotide transport and metabolism
H	164	3.77 %	165	3.86 %	Coenzyme transport and metabolism
I	117	2.69 %	106	2.48 %	Lipid transport and metabolism
P	244	5.60 %	248	5.80 %	Inorganic ion transport and metabolism
Q	77	1.77 %	69	1.61 %	Secondary metabolites biosynthesis, transport and catabolism
R	450	10.33 %	430	10.05 %	General function prediction only
S	393	9.02 %	387	9.05 %	Function unknown
-	669	15.36 %	669	15.64 %	Not in COGs

^a^The total is based on the total number of predicted protein coding genes in the annotated genomes

## Insights from the genome sequences

The genomes of the sequenced isolates were compared to the publicly available genomes of *P. agglomerans* 190 and *P. vagans* C9-1 [45] to determine the average nucleotide identity [28, 43] values between the isolates (Table 6). The ANI calculations were done with JSpecies [43] using the BLAST function, which is based on fragmenting the genomic sequence into pieces of 1,020 nucleotides long and performing similarity searches to determine homology between the genomic fragments.

**Table 6:** Average nucleotide identity (ANI) values for the sequenced isolates and additional strains representative of the lineages of *Pantoea*

	*P. agglomerans* 190	*P. agglomerans* MP2	*P. vagans* C9-1	*P. vagans* MP7	*P. anthophila* 11-2	*P. ananatis* LMG 2665	*P. stewartii* sp. *stewartii* DC283	*P. stewartii* sp. *indologenes* LMG2632	*P. dispersa* EGD-AAK13	*P. rwandensis* ND04
*P. agglomerans* 190	---	98.06	90.66	90.83	87.96	78.79	78.87	78.73	78.83	78.05
*P. agglomerans* MP2	98.75	---	91.88	91.81	89.08	79.89	79.72	79.64	79.89	78.95
*P. vagans* C9-1	90.66	91.12	---	96.62	87.56	78.79	78.81	78.75	78.75	78.1
*P. vagans* MP7	90.87	91.17	96.71	---	87.57	78.9	78.84	78.69	78.6	78.11
*P. anthophila* 11-2	88.03	88.49	87.65	87.59	---	78.97	78.9	78.72	78.92	77.93
*P. ananatis* LMG 2665	78.65	79.28	78.71	78.77	78.81	---	83.77	83.62	77.19	76.69
*P. stewartii* subsp. *stewartii* DC283	79.01	79.48	78.99	78.98	79.05	83.87	---	98.99	77.54	76.92
*P. stewartii* subsp. *indologenes* LMG2632	78.58	79.2	78.59	78.6	78.57	83.6	98.72	---	77.13	76.61
*P. dispersa* EGD-AAK13	78.68	79.35	78.69	78.64	78.85	77.3	77.37	77.27	---	82.97
*P. rwandensis* ND04	78.03	78.44	78.02	78.01	77.97	76.81	76.78	76.73	83.02	---

The number of shared genes within and between species ranged from 3,400 to 3,500. Based on the ANI values, the isolates grouped with representatives of the designated species, as species cut-off values are suggested at 95 % for ANI [28].

## Conclusion

The two bacteria described in this report were phylogenetically and genomically very closely related, but clearly belonged to different species. The ANI values supported the identification of isolates MP2 and MP7 as *P. agglomerans* and *P. vagans*, respectively.

Their similarity in genomic content may allow *P. agglomerans* and *P. vagans* to occupy the same or overlapping niches and perform the same or similar functional roles. This is consistent with what has been observed before where isolates of *P. agglomerans* and *P. vagans* occur in similar environmental niches and may even co-occur in the same environment [40]. Although recombination among micro-organisms occupying the same niche is common [3, 27], our data indicated that *P. agglomerans* and *P. vagans* have remained sufficiently distinct to identify them as separate species. This suggests that their ability to occupy the same niche is likely a function of their shared genes [13, 30, 35], but that the integrity of their individual genomic complements is protected by barriers that limit genetic exchange or gene flow between these species [14, 47].

Members of the genus *Pantoea* are often considered generalists that are isolated from a wide variety of environments [10, 19, 26]. Large metabolic repertoires (unpublished data, Marike Palmer) may allow species of this genus to form opportunistic associations with many potential hosts including insects [8, 53]. These associations, as with the biocontrol isolates [41], may be based on the *Pantoea* isolates outcompeting potentially harmful bacteria in the respective environments as microbial antagonists. This is likely also true for *P. agglomerans* and *P. vagans* and their association with termites, however recent evidence (unpublished data, Michael Poulsen) suggest that the bacterial species may provide nitrogen fixation capabilities to the termites. It is possible that the antimicrobial [21, 22, 41] and metabolic capabilities (especially pectinolytic and other carbohydrate degrading enzymes) [8] of these bacteria allow them to outcompete other, potentially harmful micro-organisms, while also providing carbohydrates and other compounds for the termites to utilize [20].
